# Classification and deep-learning–based prediction of Alzheimer disease subtypes by using genomic data

**DOI:** 10.1038/s41398-023-02531-1

**Published:** 2023-06-29

**Authors:** Daichi Shigemizu, Shintaro Akiyama, Mutsumi Suganuma, Motoki Furutani, Akiko Yamakawa, Yukiko Nakano, Kouichi Ozaki, Shumpei Niida

**Affiliations:** 1 Medical Genome Center, Research Institute, National Center for Geriatrics and Gerontology, Obu, Aichi 474-8511 Japan; 2 RIKEN Center for Integrative Medical Sciences, Yokohama, Kanagawa 230-0045 Japan; 3 Department of Cardiovascular Medicine, Hiroshima University Graduate School of Biomedical and Health Sciences, Hiroshima, 734-8553 Japan; 4 Core Facility Administration, Research Institute, National Center for Geriatrics and Gerontology, Obu, Aichi 474-8511 Japan

**Keywords:** Medical genetics, Genomics

## Abstract

Late-onset Alzheimer’s disease (LOAD) is the most common multifactorial neurodegenerative disease among elderly people. LOAD is heterogeneous, and the symptoms vary among patients. Genome-wide association studies (GWAS) have identified genetic risk factors for LOAD but not for LOAD subtypes. Here, we examined the genetic architecture of LOAD based on Japanese GWAS data from 1947 patients and 2192 cognitively normal controls in a discovery cohort and 847 patients and 2298 controls in an independent validation cohort. Two distinct groups of LOAD patients were identified. One was characterized by major risk genes for developing LOAD (*APOC1* and *APOC1P1*) and immune-related genes (*RELB* and *CBLC*). The other was characterized by genes associated with kidney disorders (*AXDND1*, *FBP1*, and *MIR2278*). Subsequent analysis of albumin and hemoglobin values from routine blood test results suggested that impaired kidney function could lead to LOAD pathogenesis. We developed a prediction model for LOAD subtypes using a deep neural network, which achieved an accuracy of 0.694 (2870/4137) in the discovery cohort and 0.687 (2162/3145) in the validation cohort. These findings provide new insights into the pathogenic mechanisms of LOAD.

## Introduction

Alzheimer’s disease (AD) is the most common cause of dementia among elderly people [1, 2]. The majority of AD cases are sporadic late-onset AD (LOAD), diagnosed in people ≥65 years [3]. The diagnosis of LOAD is characterized by the accumulation of amyloid-beta (Aβ) plaques and tau neurofibrillary tangles in the neurodegenerative brain [4], but it is a heterogeneous disease and the symptoms vary among patients. Previous studies have reported some subtypes of LOAD [5, 6]. Bredesen reported three LOAD subtypes (inflammatory, non-inflammatory, and cortical) after a metabolic profiling analysis of patients with cognitive decline [5]. Byun et al. identified four LOAD subtypes with heterogeneous patterns of regional atrophy from MRI-measured volumes of the hippocampus and cortical regions (both impaired, hippocampal atrophy only, cortical atrophy only, and both spared) [7]. However, these findings were derived from small samples, limiting their interpretation.

Genome-wide association studies (GWAS) have been highly successful in identifying genetic risk factors associated with LOAD [8]. The strongest genetic risk factor for LOAD is the apolipoprotein E ε4 (*APOE4*) polymorphism [9]. Others include bridging integrator 1 (*BIN1*) [10], clusterin (*CLU*) [10], and triggering receptors expressed on myeloid cells 2 (*TREM2*) [11], also identified from LOAD GWAS analyses. Risk prediction models using these genetic risk factors have also been developed for LOAD using various computational approaches [12, 13], including machine learning techniques [14]. With a large sample size, determining genetic risk will likely not only contribute to our understanding of the underlying pathologic mechanisms of LOAD but will also provide new insights into the classification of distinct LOAD subtypes. However, no previous studies have been conducted on genetic risks for the determination of LOAD subtypes.

Here, we comprehensively investigated the genetic architecture of LOAD based on Japanese GWAS data from a large number of patients and controls and examined the presence of LOAD subtypes using the energy landscape [15]. Each subject was represented as a binary vector based on the association signals obtained from GWAS. The energy landscape was visualized with disconnectivity graphs. We revealed the presence of two distinct groups of LOAD patients. Subsequent analysis of representative association signals from each group provided evidence that one group was characterized by both major risk genes for developing LOAD and immune-related genes, and the other group was characterized by genes associated with kidney disorders. We further found that LOAD patients had significantly decreased levels of serum albumin and hemoglobin, which are used to assess kidney function. These results suggest that impaired kidney function could be involved in LOAD pathogenesis. Our findings will contribute to a better understanding of the mechanisms driving heterogeneity in LOAD and will provide novel insight into potential subtype-specific therapies as a step toward precision medicine.

## Materials and methods

### Ethics statements

This study was approved by the ethics committee of the National Center for Geriatrics and Gerontology (NCGG). The design and performance of the current study involving human subjects were clearly described in a research protocol. All participation was voluntary, and all participants completed informed consent in writing before registering with the NCGG Biobank.

### Clinical samples

Genome-wide genotyping data from the blood samples of a total of 7284 subjects used in this study and their associated clinical data were downloaded from the NCGG Biobank database. Of the total, 4139 subjects, composed of 1947 AD subjects and 2192 cognitively normal (CN) subjects, were genotyped using the Affymetrix Japonica Array (called the discovery cohort). The remaining 3145 subjects, composed of 847 AD subjects and 2298 CN subjects, were genotyped using the Infinium Asian Screening Array (called the validation cohort). The AD subjects were diagnosed with probable or possible AD using the criteria of the National Institute on Aging and Alzheimer’s Association workgroups [16, 17]. The CN subjects had subjective cognitive complaints, but normal cognition (score >23) on a neuropsychological assessment with a comprehensive neuropsychological test, the Mini-Mental State Examination. The diagnosis of all subjects was conducted based on medical history, physical examination and diagnostic tests, neurological examination, neuropsychological tests, and brain imaging with magnetic resonance imaging or computerized tomography by experts including neurologists, psychiatrists, geriatricians, or a neurosurgeon, all experts in dementia who are familiar with its diagnostic criteria. All subjects were ≥60 years of age.

### Quality control in the GWAS

Genotype imputation was conducted by using IMPUTE2 [18] with the 3.5k Japanese reference panel developed by the Tohoku Medical Megabank Organization (https://www.megabank.tohoku.ac.jp/english/) for the discovery cohort and the 5.6k in-house reference panel for the validation set. We used imputed variants with an INFO score ≥0.4. Quality control was performed in each dataset separately after imputation using PLINK software [19]. We first applied quality control (QC) filters: (1) sex inconsistencies (--check-sex); (2) inbreeding coefficient (--het 0.1); (3) genotype missingness (--missing 0.05); (4) PI_HAT >0.25, where PI_HAT is a statistic for the proportion of identity by descent (--genome); and (5) exclusion of outliers from the clusters of East Asian populations in a principal component analysis (PCA) that was conducted together with 1000 Genomes Phase 3 data. We next applied QC filters to the genetic markers (single-nucleotide polymorphisms [SNPs] and short insertions and deletions [Indels]): (1) genotyping efficiency or call rate (--geno 0.95), (2) minor allele frequency (--freq 0.001), and (3) Hardy–Weinberg equilibrium (--hwe 0.001). To generate a set of independent SNPs, we further performed linkage disequilibrium–based SNP pruning using the statistical analysis program PLINK, version 1.90b [20] with a window size of 50 SNPs, a step of 5 SNPs, and a pairwise *r*^2^ threshold of 0.1 (--indep-pairwise 50 5 0.1).

### Disconnectivity graph

The autosomal variants that passed QC criteria as described above were assessed with a logistic regression model, adjusting for sex and age with PLINK software (--logistic) [19] in the following way:

$$logit\left( {P_i} \right) = \beta _0 + \beta _1 \times age_i + \beta _2 \times sex_i + \beta _3 \times Variant_i.$$

The coefficient and *p*-value of each variant were obtained from the discovery set. Using the variants with *p* < 0.01 weighted by their coefficients, a PCA analysis was performed. The principal component (PC) scores were binarized (i.e., −1 or +1) by the mean of the eigenvector values. Each subject was represented by a binary vector $V_k$ = ($\sigma _{1,}\sigma _{2, \cdots ,}\sigma _N$) of $2^N$, the appearance probability $P(V_k)$ of which was applied to the pairwise maximum entropy model (i.e., Boltzmann distribution). Energy values of $2^N$ binary vectors were compared to identify local energy minimums. By using a disconnectivity graph labeling the energy local minimum states, energy landscapes [15] can be visualized using the R packages *akima* and *rgl* (Supplementary Fig. 1).

### RNA-sequencing data analysis

All RNA-sequencing (RNA-seq) data were downloaded from the NCGG Biobank database [21]. The quality of the read sequences (fastq files) was assessed by using FastQC (version 0.11.7). The low-quality reads (<Q20) and trimmed reads with adapter sequences (shorter than 50 bp) were excluded by using Cutadapt (version 1.16). The remaining clean sequenced reads were mapped to the human reference genome (GRCh37) with STAR (version 2.5.2b) [22]. Quantification, in transcripts per million, was performed with RSEM (version 1.3.0).

### qRT-PCR validation of genes

cDNA was synthesized by using a PrimeScript II 1st Strand cDNA Synthesis Kit (Takara Bio, Shiga, Japan). quantitative RT-PCR (qRT-PCR) analysis was performed by using TaqMan Fast Advanced Master Mix (Thermo Fisher Scientific, Waltham, MA) and TaqMan Probes (Thermo Fisher Scientific), in accordance with the manufacturer’s instructions and the Quantstudio7 Flex Real-Time PCR System (Thermo Fisher Scientific). Target genes and their corresponding TaqMan probes were *RELB* (Hs00232399_m1) and *FBP1* (Hs00983323_m1). The qRT-PCR conditions were one cycle of 50 °C for 2 min, 95 °C for 20 s followed by 40 cycles of 95 °C for 1 s, and 60 °C for 20 s. Human beta-actin (*ACTB*, Hs99999903_m1) was preselected as a reference gene for the normalization of target gene expression levels. Gene expression levels from qRT-PCR were calculated relative to that of the reference gene *ACTB* by using the semiquantitative method [23]. Gene expression levels of *RELB* were obtained for 16 randomly selected subjects with heterozygous genotypes and 20 randomly selected subjects with homozygous genotypes. Those of *FBP* were obtained for eight randomly selected subjects with heterozygous genotypes and 20 randomly selected subjects with homozygous genotypes.

### Construction of a LOAD subtype prediction model

All data were strictly separated into a discovery cohort and a validation cohort. A PCA analysis was performed using variants with GWAS *p* < 0.01 in the discovery cohort. PC scores obtained from the PCA analysis were used to construct a disconnectivity graph. To estimate the PC scores (PC1 to PC10) from the variant data, prediction models were developed by using a deep neural network model. The neural networks comprised six hidden layers of 512 neurons along with randomized leaky rectified linear unit (RReLU) activation and 50% dropout. The networks were trained for 100 to 1500 epochs at 100 intervals with a batch size of 32 using the discovery cohort.

Estimated PC scores were binarized (i.e., −1 or +1) by the mean of eigenvector values used to construct the disconnectivity graph. Each subject was represented by a binary vector *V*_*k*_ = ($\sigma _{1,}\sigma _{2, \cdots ,}\sigma _N$) of $2^N$, from which the position on the disconnectivity graph was detected. Prediction models were assessed by the rate at which LOAD cases and CN subjects were predicted in the red and green spheres of the same groups on the disconnectivity graph. The LOAD subtype prediction models were evaluated on the independent validation cohort. These models were constructed using the open-source machine learning library PyTorch (version 1.7.0).

## Results

### GWAS of the Japanese subjects

The discovery cohort consisted of 1947 LOAD cases and 2192 CN subjects. The average ages of the subjects from whom the LOAD and CN blood samples were obtained was 79.1 years (SD = 6.3 years) and 70.8 years (SD = 5.9 years), respectively, and the female-to-male ratios were 2.13:1 and 1.19:1, respectively. We examined association signals from the discovery cohort. A total of 9,252,465 genetic markers (SNPs and Indels) passed stringent QC filters for both genotypes and samples after genotype imputation. The QC-passed genetic markers were further pruned for linkage disequilibrium to obtain a set of independent markers. A subset of 2,741,829 genetic markers was used for subsequent analysis.

The validation cohort consisted of 847 LOAD cases and 2298 CN subjects. The average ages of the subjects from whom the LOAD and CN samples were obtained was 79.4 years (SD = 6.4 years) and 75.4 years (SD = 4.3 years), respectively, and the female-to-male ratios were 1.91:1 and 1.27:1, respectively.

### Disconnectivity graph construction

**Fig. 1:**
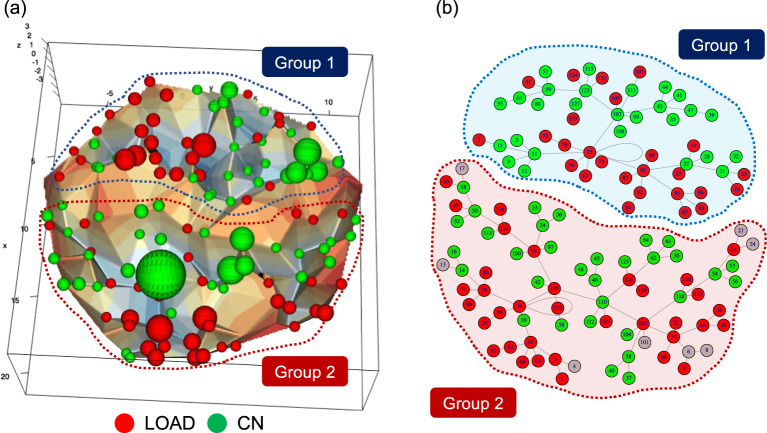
Disconnectivity graph. The energy landscape was visualized by using 3D (**a**) and 2D (**b**) disconnectivity graphs, where all samples were classified into two groups. LOAD cases and CN subjects are represented in red and green spheres, and their sizes correspond to the difference of LOAD and CN frequencies at each node. **b** Gray circles represent to the same frequency in LOAD and CN subjects.

Of the 2,741,829 genetic markers from the discovery cohort, 22,128 showed association signals with GWAS *p* < 0.01. Using the association signals, we performed a PCA. According to the PC scores, subjects were binarized to 2^7^ binary vectors. Local energy minimum states were obtained from a comparison of energy values among the binary vectors, and the energy landscape was visualized with disconnectivity graphs labeling their local energy minimum states. In Fig. 1a, LOAD cases and CN subjects are represented as red and green spheres whose sizes correspond to the difference in LOAD and CN frequencies (i.e., the red spheres represent that LOAD cases are more frequent than controls and the green spheres represent that CN subjects are more frequent than LOAD cases). Both LOAD cases and CN subjects created clusters, and we revealed the presence of two distinct groups in LOAD and in CN (Fig. 1b).

### Demographic data of the two LOAD groups

The discovery cohort was classified into two distinct groups: group 1 was composed of 918 AD cases and 895 CN subjects, and group 2 contained 1029 AD cases and 1297 CN subjects. The demographic data of the subjects in each group are shown in Table 1. No differences were observed between the two groups in the average age and female-to-male ratio, but statistically significant differences were found in the number of *APOE4* alleles between the LOAD subjects in each group (Fisher’s exact test *p* = 3.70 × 10^−12^) and between the CN subjects in each group (Fisher’s exact test *p* = 1.06 × 10^−8^), with the proportion of subjects with *APOE4* higher in group 1 than in group 2. When visualizing the energy landscape by *APOE4* and *APOE2* information, significant positive correlations between LOAD frequency and *APOE4* frequency were observed, with a higher correlation coefficient in group 1 (Kendall’s *τ* = 0.48, *p* = 6.72 × 10^−7^) than in group 2 (Kendall’s *τ* = 0.41, *p* = 1.59 × 10^−6^) (Supplementary Fig. 2).

**Table 1:** Clinical characteristics of the discovery cohort.

Group	Factor	LOAD	CN
1	Number of subjects	918	895
	Age (y; mean ± 1 SD)	78.87 ± 5.96	70.38 ± 5.67
	% Female (#subjects)	68.52 (629)	54.08 (484)
	*#APOE4* alleles (#subjects)	0 (457), 1 (383), 2 (78)	0 (678), 1 (206), 2 (11)
	% Subjects with *APOE4*	50.22	24.26
2	Number of subjects	1029	1297
	Age (y; mean ± 1 SD)	79.29 ± 6.53	71.10 ± 5.97
	% Female (#subjects)	67.54 (695)	54.43 (706)
	*#APOE4* alleles (#subjects)	0 (674), 1 (311), 2 (44)	0 (1112), 1 (179), 2 (6)
	% Subjects with *APOE4*	34.50	14.26

*LOAD* late-onset Alzheimer’s disease, *CN* cognitively normal.

### Representative association signals of each group and their functional annotations

To identify distinctive association signals in each group, we focused on representative clusters where LOAD and CN frequencies differed by more than 5% (i.e., the red or green spheres in Fig. 1 were large). We observed six representative clusters (three red and three green spheres) in group 1, which contained 20,792 genetic markers from 848 subjects (391 LOAD cases and 457 CN samples), and five representative clusters (two red and three green spheres) in group 2, which contained 19,929 genetic markers from 1047 subjects (305 LOAD cases and 742 CN samples). Association signals were detected with logistic regression, adjusting for sex and age. From group 1, four genetic markers, located on or near four genes (*APOC1*, *APOC1P1*, *RELB*, and *CBLC*) showed statistically significant associations at Bonferroni-corrected *p* < 0.05; from group 2, 12 genetic markers, located on or near four genes (*AXDND1*, *FBP1*, *AOPEP*, and *MIR2278*) had significant associations (Table 2). Group 1 was characterized by major risk genes for developing LOAD (*APOC1* and *APOC1P1*) [24] and immune-related genes (*RELB* and *CBLC*) [25–27], whereas group 2 was characterized by genes associated with kidney disorders, such as nephrotic syndrome (*AXDND1, FBP1*, and *MIR2278*) [28, 29]. Our results were consistent with previous findings of associations between kidney disease and AD [30] and between the immune system and AD [31].

**Table 2:** Association signals detected with logistic regression.

Group	Chromosome: Position	SNP	Allele 1/2	Closest gene	Odds Ratio	95% CI	p*	Bonferroni corrected *p*
1	19: 45416478	rs584007	G/A	*APOC1*	2.07	1.60-2.67	2.53 × 10^−8^	4.56 × 10^-4^
	19: 45428318	rs149000543	T/C	*APOC1P1*	14.30	5.15-39.74	3.38 × 10^−7^	6.09 × 10^-3^
	19: 45536170	**rs146190016**	C/T	***RELB***	7.61	3.36-17.23	1.15 × 10^−6^	0.021
	19: 45299058	rs141177830	T/C	*CBLC*	4.51	2.40-8.44	2.64 × 10^−6^	0.048
2	1: 179475274	rs76860468	T/C	*AXDND1*	0.097	0.038-0.25	1.32 × 10^−6^	0.023
	1: 179489735	rs76862983	A/C	*AXDND1*	0.097	0.038-0.25	1.32 × 10^−6^	0.023
	1: 179430794	rs141737442	A/G	*AXDND1*	0.097	0.038-0.25	1.42 × 10^−6^	0.025
	9: 97393347	**rs550833079**	G/A	***FBP1***	26.30	6.80-101.7	2.14 × 10^−6^	0.038
	9: 97505339	rs370031304	T/C	*AOPEP*	58.88	10.87-319	2.28 × 10^−6^	0.041
	9: 97516428	rs373357289	G/A	*AOPEP*	58.88	10.87-319	2.28 × 10^−6^	0.041
	9: 97527474	rs540479525	A/G	*AOPEP*	58.88	10.87-319	2.28 × 10^−6^	0.041
	9: 97561445	rs117828945	C/T	*AOPEP*	58.88	10.87-319	2.28 × 10^–6^	0.041
	9: 97575958	rs147862129	T/G	*MIR2278*	58.88	10.87-319	2.28 × 10^−6^	0.041
	9: 97593604	rs112195629	G/A	*MIR2278*	58.88	10.87-319	2.28 × 10^−6^	0.041
	9: 97609459	rs369354585	G/GTCTC	*MIR2278*	58.88	10.87-319	2.28 × 10^−6^	0.041
	9: 97626803	rs193212269	A/G	*MIR2278*	58.88	10.87-319	2.28 × 10^−6^	0.041

^*^Linear regression, adjusting for sex and age.

Bold, association signals of *RELB* and *FBP1* (rs146190016 and rs550833079) were located within their genes.

### Closest gene expression of association signals using blood samples

**Fig. 2:**
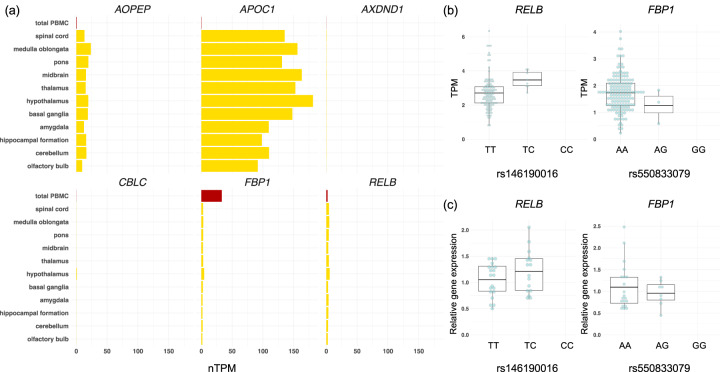
Validation of expression of the genes closest to the association signals using qRT-PCR. **a** The expression of six genes in the blood (red) and brain tissues (yellow) was checked in the HPA database. The *x*-axis represents the resulting transcript expression values, denoted as normalized transcripts per million (nTPM). **b** RNA-seq data of 126 subjects and 151 subjects were available from the NCGG Biobank database for *RELB* and *FBP1*, respectively. The effects of association signals on the expression of their nearby genes were examined by using linear regression adjusted for age and sex. The association signal rs146190016 significantly increased *RELB* expression (*p* = 0.019), but the signal rs550833079 did not significantly change *FBP1* expression (*p* = 0.37). **c** We used quantitative RT-PCR (qRT-PCR) data to validate eQTL results for these genes (*RELB* and *FBP1*). The results were consistent with the RNA-seq results for both genes. Data in (**b**) and (**c**) are represented as box and whisker plots, depicting minimum, lower quartile (Q1), mean (Q2), upper quartile (Q3), and maximum values.

We used blood samples to examine whether the association signals detected affect expression of their closest genes. Of the eight genes, the Human Protein Atlas (HPA) database (https://www.proteinatlas.org) indicated that two (*RELB* = 2.0 and *FBP1* = 33.1) are expressed in blood cells, where expressed genes were defined as genes with normalized transcripts per million ≥2 in total human peripheral blood mononuclear cells. Although *MIR2278* and *APOC1P1* have not been registered in the HPA database, the HPA database showed that *APOC1*, *AOPEP, RELB*, and *FBP1* are expressed in brain tissues (Fig. 2a).

Association signals of *RELB* and *FBP1* (rs146190016 and rs550833079) were located within their genes. The whole-blood RNA-seq data are available from the NCGG Biobank database. We examined the effect of the association signals on the expression of these genes by using linear regression adjusting for age and sex. We downloaded data on *RELB* for 126 of the 848 subjects in group 1 and data on *RBP1* for 151 of the 1047 subjects in group 2 from the NCGG Biobank database. Although rs550833079 was not associated with a significant expression change in *FBP1* (*p* = 0.37), rs146190016 contributed to a statistically significant increase of *RELB* expression (*p* = 0.019, Table 2 and Fig. 2b). To validate the expression Quantitative Trait Locus (eQTL) results for these genes, we used qRT-PCR data, which were consistent with the RNA-seq results for both genes (Fig. 2c).

### Assessment of kidney function

**Fig. 3:**
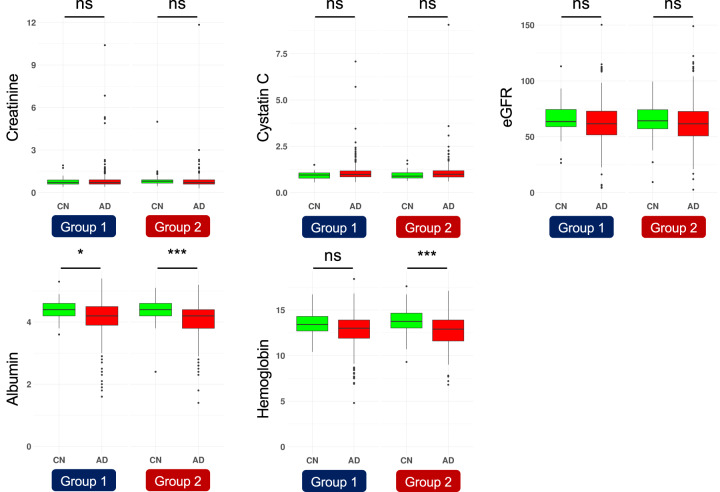
Assessment of kidney function using routine blood test results. We examined five markers of kidney function measured in routine blood tests (creatinine, cystatin C, eGFR, albumin [Alb], and hemoglobin [Hb]). The differences in the results between LOAD and CN were tested with the Wilcoxon rank-sum test. Data were represented as box and whisker plots, depicting minimum, lower quartile (Q1), mean (Q2), upper quartile (Q3), and maximum values. *FDR < 0.05, **FDR < 0.001.

Five measurements for assessment of kidney function (creatinine, cystatin C, eGFR, albumin [Alb], and hemoglobin [Hb]) were measured in routine blood tests. We obtained these data for 387 (342 LOAD cases and 45 CN subjects) from group 1 and 355 (269 LOAD cases and 86 CN subjects) from group 2. The difference in the measurements between LOAD and CN was determined with the Wilcoxon rank-sum test. We found that albumin and hemoglobin reached a false discovery rate (FDR) of significance (FDR_Alb_ = 0.012 in group 1; FDR_Alb_ = 1.30 × 10^−6^, FDR_Hb_ = 4.53 × 10^−6^ in group 2, Fig. 3), although the remaining three measures showed no statistically significant difference between LOAD and CN in either group. These results support the hypothesis that impaired kidney function can lead to LOAD pathogenesis.

As type 2 diabetes mellitus (T2D) is known to increase the risk for dementia, including AD [32], we examined the level of HbA1c measured in routine blood tests. We obtained the data for 388 (343 LOAD cases and 45 CN subjects) from group 1 and 355 (269 LOAD cases and 86 CN subjects) from group 2. The difference in levels between LOAD and CN was determined with the Wilcoxon rank-sum test, but there were no statistically significant differences between LOAD and CN subjects in both groups (*P* = 0.687 in group 1; *P* = 0.696 in group 2, Supplementary Fig. 3).

### LOAD subtype prediction model

**Fig. 4:**
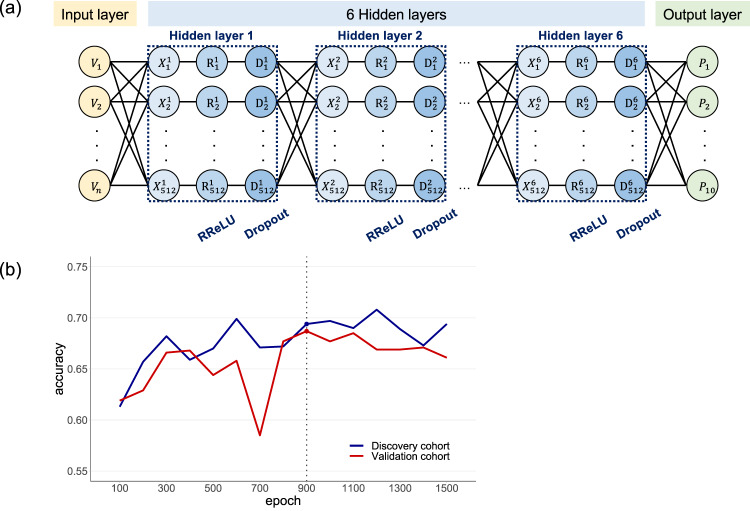
A LOAD subtype prediction model. **a** We applied a deep neural network with six hidden layers of 512 neurons along with RReLU activation, 50% dropout, and a batch size of 32 to predict PC scores from variant data of the discovery cohort. **b** The networks were trained for 100 to 1500 epochs at 100 intervals using the discovery cohort. The best model achieved an accuracy of 0.694 in 900 epochs in the discovery cohort and 0.687 in the independent validation cohort.

A disconnectivity graph was constructed based on PC scores, obtained from the PCA analysis using 22,128 variants with GWAS *p* < 0.01 in the discovery cohort. Of them, 17,743 variants were common with those in the independent validation cohort (see the Methods). The genomic variants could predict LOAD subtypes of patients. We applied a deep neural network with six hidden layers of 512 neurons along with RReLU activation, 50% dropout, and a batch size of 32 to predict PC scores from variant data of the discovery cohort (Fig. 4a). The predicted PC scores were binarized by the mean of eigenvector values used for the construction of the disconnectivity graph. Each subject was represented by a binary vector $V_7$ = ($\sigma _{1,}\sigma _{2, \cdots ,}\sigma _7$) of 2^7^, from which the location on the disconnectivity graph was determined. Prediction models were assessed by the rate at which LOAD cases and CN subjects were predicted in the red and green spheres of the same groups on the disconnectivity graph. We constructed neural network models with several hidden layers (Layer = 1, 2, …,7) and assessed them using the independent validation cohort (Supplemental Fig. 4). Our LOAD subtype prediction model (i.e., a deep neural network model comprised of six hidden layers) achieved an accuracy of 0.694 (2870/4137) in 900 epochs in the discovery cohort (group 1, 1228/1812 = 0.678; group 2, 1642/2325 = 0.706) and of 0.687 (2162/3145) in the validation cohort (group 1, 1025/1508 = 0.680; group 2, 1137/1637 = 0.695, Fig. 4b).

## Discussion

Using the genotyping data from a case-control cohort of >4100 subjects, we examined genetic risks for the determination of LOAD subtypes and revealed the presence of two distinct groups of LOAD patients. Clinical characteristics of the groups showed a significant difference in the frequency of *APOE4* alleles, although no differences were observed in the average age and the female-to-male ratio between groups. To understand the genetic difference between groups, we further investigated representative association signals in each group. Four representative genes were detected from each group: group 1, *APOC1*, *APOC1P1*, *RELB*, and *CBLC*; group 2, *AXDND1*, *FBP1*, *AOPEP*, and *MIR2278*.

The four genes in group 1 (*APOC1*, *APOC1P1*, *RELB*, and *CBLC*) are located near the *APOE* region, and *APOC1* and *APOC1P1* are in linkage disequilibrium with the *APOE* gene. However, previous studies have shown that they are *APOE*-independent risk factors for AD [24]. *RELB* is a member of the nuclear factor kappa B (NF-κB) family [25]. Activated NF-κB has been found in neurons and glial cells in the brains of AD patients [33]. *CBLC* (Cbl proto-oncogene C) is a member of the Cbl family of E3 ubiquitin ligases. Recent studies have shown that *CBLC* is likely to indirectly modulate the stimulatory function of NF‐*κ*B complexes [34]. Nho et al. reported that *RELB* and *CBLC* were significantly associated with LOAD-related cerebrospinal fluid Aβ_1–42_ with adjustment for *APOE* genotype [35]. These results suggest that the representative genes in group 1 are *APOE*-independent risk factors for LOAD and are likely to contribute to Aβ accumulation through direct or indirect NF‐*κ*B activation.

On the other hand, three of the four representative genes in group 2 (*AXDND1*, *FBP1*, and *MIR2278*) are likely to be associated with kidney diseases. Several variants in *AXDND1* have been registered as mutations involved in nephrotic syndrome in the GeneCards [28] and ClinVar databases [36]. *FBP1* (fructose-bisphosphatase1) is a gluconeogenesis regulatory enzyme, which is suppressed in kidney tumors [37]. Although *AOPEP* has not been shown to be associated with kidney diseases, *MIR2278* (microRNA 2278) can target *STAT5A* [38], which has been reported to contribute to the pathogenesis of kidney disease [39]. We further examined markers for kidney function measured in routine blood test results. Statistically significant differences between LOAD and CN were found in albumin and hemoglobin concentrations. These results suggest that impaired kidney function in group 2 LOAD patients could have led to AD pathogenesis. Wu et al. reported that AD severity was significantly associated with serum albumin and hemoglobin [40].

In this study, we revealed the presence of two distinct groups of LOAD patients through the genetic architecture of LOAD based on Japanese GWAS data, clearly revealing the presence of LOAD subtypes. We also used a deep neural network to create a LOAD subtype prediction model that achieved high accuracies in the discovery and validation cohorts, as our model can predict LOAD subtypes and phenotype simultaneously (i.e., LOAD group 1, LOAD group 2, CN group 1, and CN group 2). We believe that our findings will contribute to the understanding of LOAD and provide new insights into its pathogenic mechanisms. Many clinical trials using anti-Aβ antibody drugs, even Lecanemab, have not shown convincing results. This might be due to the existence of LOAD subtypes with different genetic backgrounds. Additional association studies between LOAD and CN using whole-genome sequencing data from large samples including non-Japanese will undoubtedly lead to further understanding of LOAD subtypes in the future.

## Supplementary information

Figure S1

Figure S2

Figure S3

Figure S4

## Data Availability

All GWAS summary datasets used or analyzed in the current study are available from the corresponding author on reasonable request.

## Code availability

We used open-source program languages R (version 3.4.3) and Ruby (version 2.4.0) and the machine learning library PyTorch (version 1.7.0) to analyze data and create plots. Code is available upon request from the corresponding author.
