# Supplementary material for: Classification and deep-learning–based prediction of Alzheimer disease subtypes by using genomic data
Source: Transl Psychiatry. 2023 Jun 29;13:232. doi: 10.1038/s41398-023-02531-1 (PMC10310810; doi:10.1038/s41398-023-02531-1)
Supplement: Supplementary file 1 — Figure S1 [file 41398_2023_2531_MOESM1_ESM.pdf]

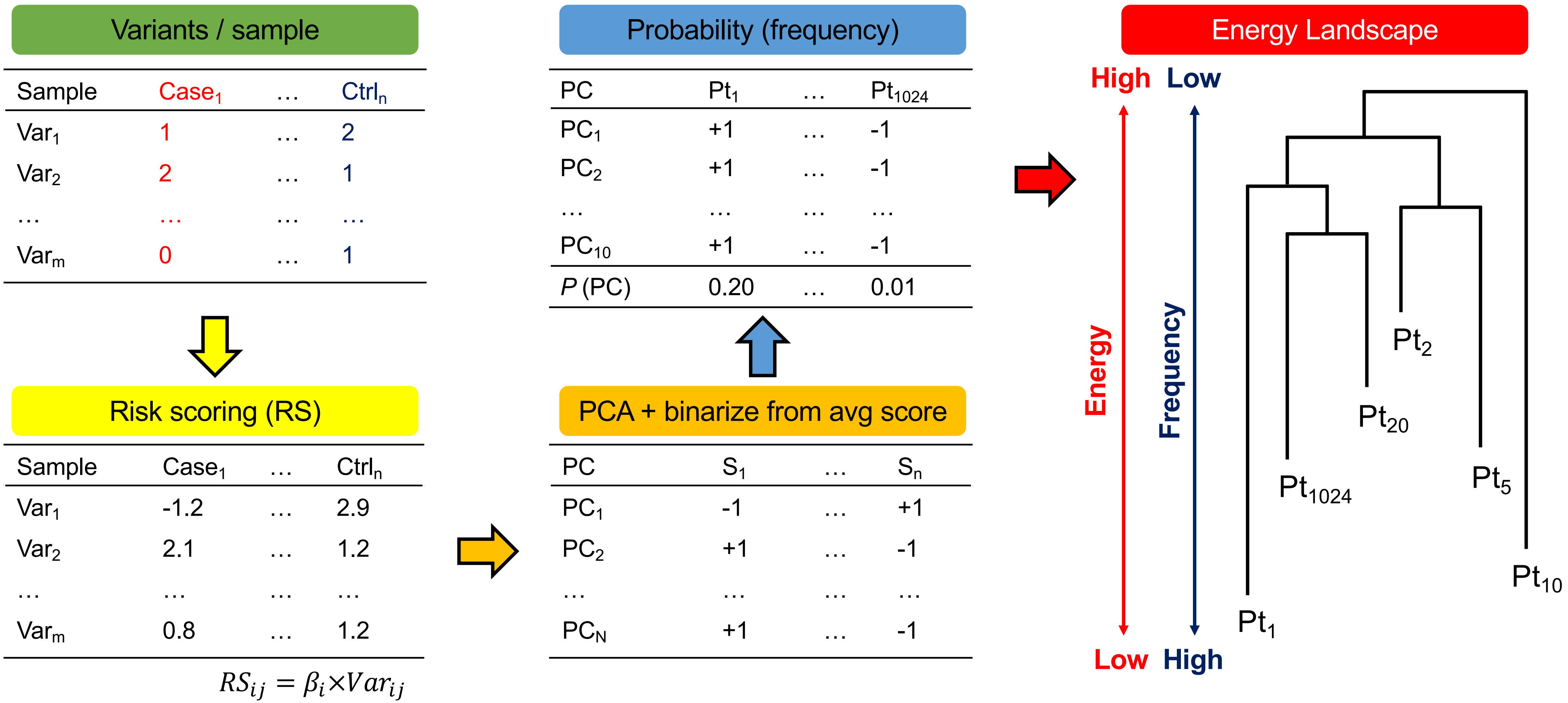

Supplementary Figure 1. Outline of energy landscape

The autosomal variants that passed QC criteria were assessed with a logistic regression model, adjusting for sex and age. Using the variants with $p < 0.01$ weighted by their coefficients, a PCA analysis was performed. The principal component (PC) scores were binarized (i.e., −1 or +1) by the mean of the eigenvector values. Each subject was represented by a binary vector of 2^N. Energy values of 2^N binary vectors were compared to identify local energy minimums. By using a disconnectivity graph labeling the energy local minimum states, energy landscapes can be visualized using the R packages.
