# Supplementary material for: Classification and deep-learning–based prediction of Alzheimer disease subtypes by using genomic data
Source: Transl Psychiatry. 2023 Jun 29;13:232. doi: 10.1038/s41398-023-02531-1 (PMC10310810; doi:10.1038/s41398-023-02531-1)
Supplement: Supplementary file 2 — Figure S2 [file 41398_2023_2531_MOESM2_ESM.pdf]

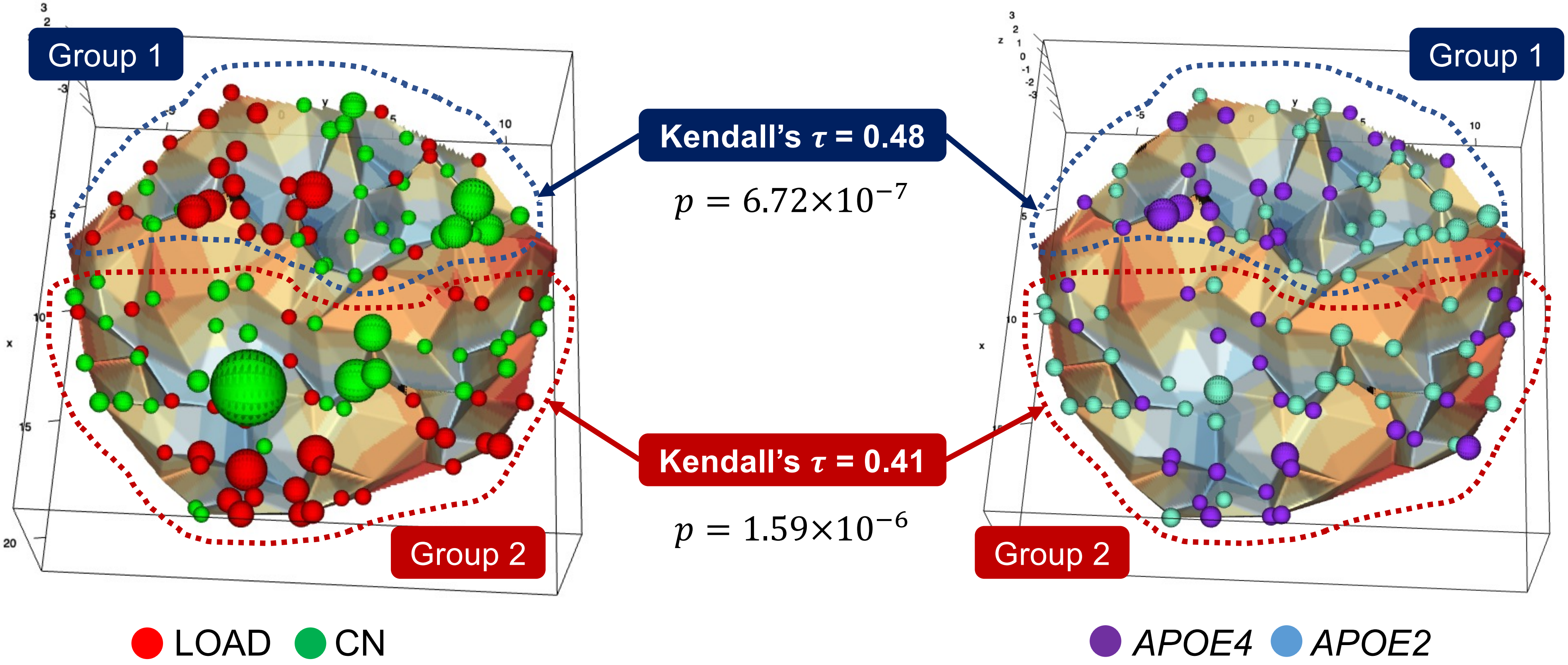

Supplementary Figure 2. Visualization of the energy landscape by *APOE4* and *APOE2* information

LOAD frequency and *APOE4* frequency were significantly correlated, with a higher correlation coefficient in group 1 (Kendall's τ = 0.48, p = 6.72 $\times 10^{-7}$) than in group 2 (Kendall's τ = 0.41, p = 1.59 $\times 10^{-6}$).
