# Supplementary material for: Classification and deep-learning–based prediction of Alzheimer disease subtypes by using genomic data
Source: Transl Psychiatry. 2023 Jun 29;13:232. doi: 10.1038/s41398-023-02531-1 (PMC10310810; doi:10.1038/s41398-023-02531-1)
Supplement: Supplementary file 3 — Figure S3 [file 41398_2023_2531_MOESM3_ESM.pdf]

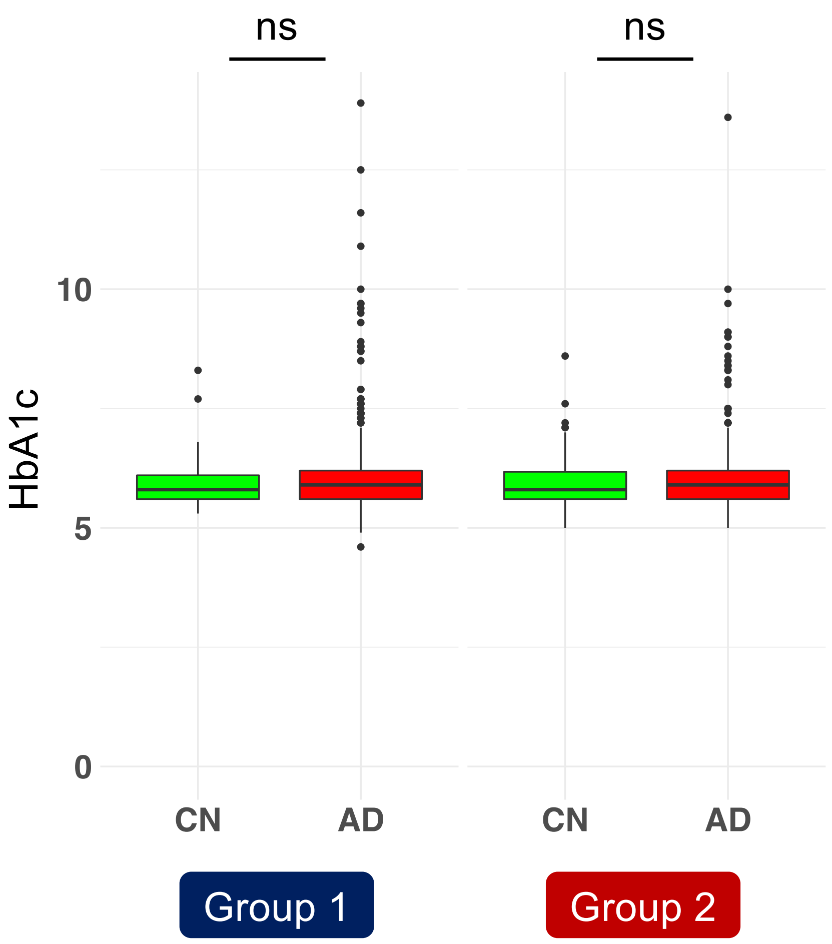

Supplementary Figure 3. Assessment of HbA1c levels.
We examined HbA1c measured in routine blood tests. The differences of the results between LOAD and CN were tested with the Wilcoxon rank sum test. Data are represented as box and whisker plots, depicting minimum, lower quartile (Q1), mean (Q2), upper quartile (Q3), and maximum values.
