# Supplementary material for: Classification and deep-learning–based prediction of Alzheimer disease subtypes by using genomic data
Source: Transl Psychiatry. 2023 Jun 29;13:232. doi: 10.1038/s41398-023-02531-1 (PMC10310810; doi:10.1038/s41398-023-02531-1)
Supplement: Supplementary file 4 — Figure S4 [file 41398_2023_2531_MOESM4_ESM.pdf]

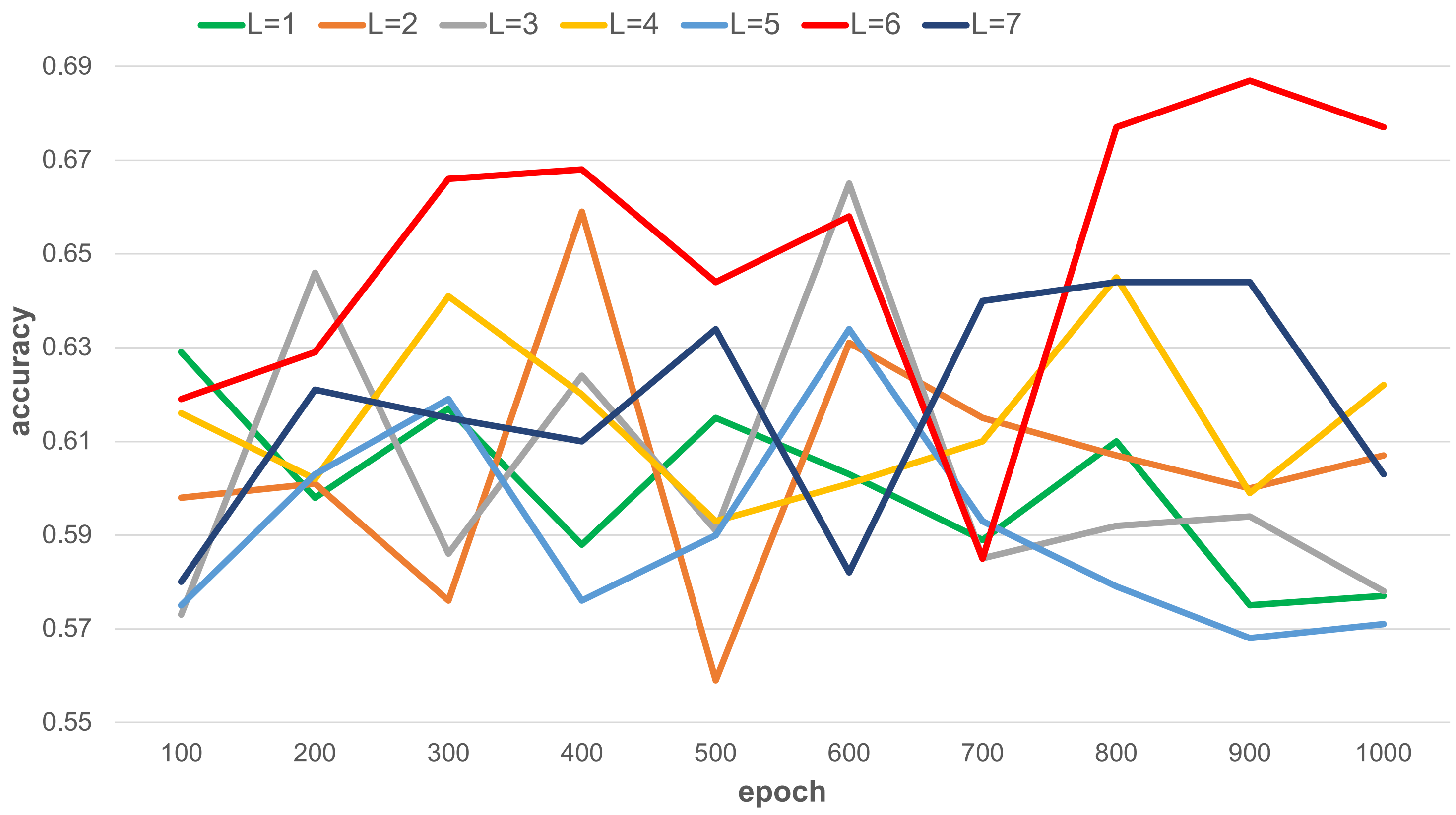

Supplementary Figure 4. LOAD subtype prediction models with a deep neural network
We applied a deep neural network with several hidden layers (Layer; L=1, 2, …7) of 512 neurons along with RReLU activation, 50% dropout, and a batch size of 32 to predict PC scores from variant data of the discovery cohort. The networks were trained for 100 to 1000 epochs at 100 intervals using the independent validation cohort.
